# Housing-based syringe services programs to improve access to safer injecting equipment for people who inject drugs in Vancouver, Canada: a spatially oriented qualitative study

**DOI:** 10.1186/s12954-023-00862-2

**Published:** 2023-09-07

**Authors:** Koharu Loulou Chayama, Cara Ng, Taylor Fleming, Will Small, Kimberly L. Sue, Ryan McNeil

**Affiliations:** 1 British Columbia Centre on Substance Use, 1045 Howe Street, Vancouver, Canada; 2 Interdisciplinary Studies Graduate Program, University of British Columbia, 6371 Crescent Road, Vancouver, Canada; 3 Faculty of Health Sciences, Simon Fraser University, 8888 University Drive, Burnaby, Canada; 4 Program in Addiction Medicine, Yale School of Medicine, New Haven, CT 06520 USA; 5 Department of Internal Medicine, Yale School of Medicine, 333 Cedar Street, New Haven, USA

**Keywords:** Housing, Injection drug use, People who inject drugs, Syringe services programs

## Abstract

**Background:**

Housing environments shape injection drug-related risks and harms and thus represent a critical implementation setting for syringe services programs (SSPs). As critical harm reduction measures, SSPs provide safe injecting equipment to people who inject drugs (PWID). Vancouver, Canada, has well-established syringe distribution programs through which PWID have low-threshold access to unlimited syringes and related injecting equipment, including through non-profit operated supportive housing and single-room occupancy hotels. This study examines the role of housing-based SSPs in distributing injecting equipment to PWID in Vancouver.

**Methods:**

Between January and March 2020, semi-structured, in-depth interviews were conducted in Vancouver with 26 PWID. Interviews were audio-recorded, transcribed, and coded. Salient themes were identified using inductive and deductive approaches.

**Results:**

Many participants accessed SSPs in housing facilities and expressed preference for these programs over those offered at other locations and through other health and social services. Three major themes emerged to explain this preference. First, most participants injected in the buildings where they resided, and housing-based SSPs made injecting equipment available when and where it was most needed. Second, many participants preferred to avoid carrying syringes outside of the places where they inject due to fears that syringe possession may lead to criminal charges or confiscation of syringes and/or illicit drugs by police. Third, for some participants, anti-drug user stigma and concerns over unwillingly disclosing their drug use hindered access to SSPs outside of housing settings. Programs operated within housing facilities often offered greater client anonymity along with more supportive and less stigmatizing environments, particularly in the presence of peer staff.

**Conclusion:**

The current study advances understanding of access to injecting equipment in a setting with city-wide syringe distribution programs. Our findings underscore the benefits of housing-based SSPs and encourage the expansion of such services to maximize access to harm reduction supports for PWID.

## Background

Housing environments have long been demonstrated to shape health [1–5]. Housing environments that can be characterized as marginalized on the basis of substandard physical conditions and management practices, such as single-room occupancy hotels (SROs), have been linked to poor health outcomes including fatal overdose and transmission of HIV, hepatitis C (HCV), and other blood-borne infections among people who inject drugs (PWID) [1–5]. In cities across North America, SROs serve as the “housing of last resort” for structurally vulnerable populations such as PWID [1]. With the persistent lack of alternate spaces to use illicit drugs, SROs are often used as private indoor spaces where PWID can use drugs, either alone or in groups, with reduced risks of criminalization and stigmatization compared to public and semi-public settings [3]. Research on the implementation of harm reduction interventions, such as syringe services programs (SSPs), has shed light on how environmental changes can reduce drug-related harms for PWID [6]. Higher-risk housing environments, such as SROs, thus represent a critical implementation setting for these interventions.

SSPs are evidence-based harm reduction interventions that provide free safer injecting equipment for PWID, including sterile syringes and needles, as well as other injecting equipment such as sterile water, cookers, filters, ascorbic acid, alcohol swabs, and tourniquets [6]. Over three decades of research has established their cost-effectiveness and effectiveness in reducing syringe sharing associated with the transmission of HIV, HCV, and other blood-borne infections [6]. These programs have been endorsed by the World Health Organization and the Joint United Nations Program on HIV/AIDS, among other local, national, and international health organizations [7, 8]. SSPs can be stand-alone or integrated within existing services that serve PWID, and provide injecting equipment through a range of service delivery models [9, 10]. Some models such as vending machines and mobile van outlets have been identified as particularly effective at removing barriers and facilitating access to injecting equipment for structurally vulnerable populations [11]. However, there remain significant gaps in coverage of injecting equipment to meet needs and high rates of syringe sharing have persisted in some settings [12–14].

Due to the misperception that SSPs “enable” drug use and the ongoing stigma against drug use and PWID, many SSPs still operate under restrictive policies, such as unitary one-for-one exchange policies, in which clients are required to return their used syringes to receive an equal number of new syringes [15]. Such policies limiting the number of syringes distributed are widely acknowledged as unsatisfactory by harm reductionists and contrary to the research evidence on SSP models [16]. Driven by community organizing and research demonstrating effectiveness [17–19], programs that operate under a needs-based distribution policy, where clients can access as many syringes as they want without the expectation that they be returned, have been established as public health best practice for SSPs [16, 20].

Vancouver, Canada, is a site of one of the world’s largest syringe distribution programs whose service delivery model has evolved in response to community organizing and research evidence [18, 21, 22]. To maximize coverage of injecting equipment for Vancouver’s 15,000 PWID, of whom 23% were estimated to be living with HIV by the late 1990s [2], the city’s SSP policy was modified by the local health authority in the early 2000s [22]. Building on the low-threshold policy pioneered by the Vancouver Area Network of Drug Users (VANDU), a local drug user organization [19], the one-for-one exchange policy was eventually replaced by a needs-based distribution policy [22]. In addition to removing the restriction on the number of syringes that could be distributed at one time, the changes involved increasing the number and variety of sites distributing injecting equipment [22]. Addressing drug-related harms associated with high-risk housing environments that had long been identified as an issue by local researchers [23, 24], as well as non-profit housing providers [25], this policy shift expanded the availability of SSPs in housing facilities such as non-profit operated supportive housing and SROs [22]. Changes in SSP policy during this time have been associated with substantial declines in rates of syringe sharing and HIV incidence among PWID in Vancouver [22].

While it has been suggested that housing-based SSPs may be critical to mitigating drug-related harms in housing environments, little research attention has been paid to how these programs are experienced and situated within the everyday lives and geographies of PWID. Thus, we undertook this spatially oriented qualitative study to explore the role of housing-based SSPs in distributing injecting equipment to PWID in Vancouver. Greater understanding of housing-based SSPs will be essential to informing efforts to maximize syringe coverage and improving access to harm reduction supports for PWID.

## Methods

We draw upon semi-structured, in-depth interviews and mapping exercises conducted with 26 PWID in Vancouver from January to March 2020 (when research activities were suspended by the COVID-19 pandemic). Given that “place” is a key determinant of health among PWID [26], we adopted a spatially oriented qualitative approach by linking narrative data from interviews with geospatial data from mapping exercises to examine social, structural, and spatial influences on access to injecting equipment, with a particular focus on PWID’s experiences with housing-based SSPs.

Housing-based SSPs provide injecting equipment to PWID, including unlimited sterile syringes and needles, as well as other injecting equipment such as sterile water, cookers, filters, ascorbic acid, alcohol swabs, and tourniquets. In Vancouver, housing-based SSPs typically operate out of non-profit operated supportive housing and SROs, and have extensive hours of operation (e.g., 24 h a day and 7 days a week). Injecting equipment is usually offered in the lobby, often at the front desk, and is either distributed by building staff or freely available to residents. Non-residents may be given access to housing-based SSPs—although, this may not be the case everywhere due to variations in building policies (e.g., no-guest policies). Alongside injecting equipment, housing facilities that operate an SSP offer sharps disposal containers. They may also offer other harm reduction supports (e.g., naloxone kits, overdose response button technology). Note that there is no formalized policy for operating housing-based SSPs in Vancouver. Rather, available housing-based harm reduction services are intended to be implemented to meet the needs of each specific building [27], and thus, housing-based SSPs may look different across our participant sample.

This study was conducted in connection with two prospective cohort studies of people who use drugs: the AIDS Care Cohort to evaluate Exposure to Survival Services (ACCESS) and Vancouver Drug Users Study (V-DUS). For both cohorts, individuals who were 18 years of age or older and live in Greater Vancouver were recruited through word of mouth, street outreach, and referrals [2, 28]. Participants were eligible for ACCESS if they were living with HIV and used illicit drugs other than cannabis in the previous month [28]. Participants were eligible for V-DUS if they were not living with HIV and injected or smoked drugs in the previous month. These cohorts are described in greater detail elsewhere [2, 28]. Participants enrolled in these cohort studies were screened for eligibility by cohort study staff during their routine cohort study interviews and, if they expressed interest in participating, were scheduled for a qualitative interview and mapping exercise. For the current study, data from participants who reported injecting drugs in the last 30 days were examined. The study was approved by the University of British Columbia/Providence Health Care Research Ethics Board.

Interviews were conducted in a private room at a storefront research office located in Vancouver’s Downtown Eastside (DTES) neighborhood, home to Canada’s largest street-based drug scene. Prior to each interview, interviewers provided participants with an explanation of the study, answered any questions, and obtained written informed consent. An interview guide was used to facilitate discussion on topics related to current life situation (e.g., living arrangements, income generation, police encounters), drug use and risk behaviors (e.g., syringe sharing), and access to harm reduction supplies and services (e.g., safer injecting equipment and SSPs; see Table 1 for an abbreviated version of the interview guide). Interviews involved mapping exercises during which participants identified locations key to their everyday spatial practices on a physical map of Vancouver. The interview guide was developed based on a review of the relevant literature and by drawing on the experiences of the research team. Interviews were approximately 30–60 min in length and audio-recorded. Upon completion of the interviews, participants received an honorarium ($30 CAD) as compensation for their time. Interviews were transcribed by a professional transcription service and reviewed for accuracy by the interviewers.

**Table 1:** Abbreviated interview guide

Questions	Mapping exercise
	Indicate locations of the following on a map:
*Current life situation*	
Where are you currently staying?	Primarily stayed Stayed occasionally
How do you generate income? Where do you work?	Primarily worked Worked occasionally
Have you ever had an encounter with the police?	Encountered police Arrested by police
*Drug use and risk behaviors*	
How would you describe your drug use? Where do you buy drugs? Use drugs? Dispose needles/syringes and other equipment?	Bought drugs Used drugs Disposed safer injecting equipment
Can you walk me through the process of a typical time that you might use drugs in the last month?	
Do you ever reuse or share safer injecting equipment?	
How and when do you dispose safer injecting equipment?	
*Access to harm reduction supplies and services*	
Where do you get safer injecting equipment?	Accessed safer injecting equipment
When, where and how often do you currently access safer injecting equipment?	
Why do you access safer injecting equipment in these places?	
Do you feel that there are any advantages or disadvantages to accessing safer injecting equipment in these places? If so, what and why?	
Where do you prefer to access safer injecting equipment?	

Data were analyzed using a qualitative GIS approach involving techniques integrating qualitative and geospatial data within NVivo qualitative analysis software and QGIS geographic information software [29]. Geospatial data from the mapping exercises were imported into QGIS to produce digital maps, including aggregate maps depicting the distribution of harm reduction services and police encounters, and individual maps of locations of importance. Interview transcripts and maps were then imported into NVivo to facilitate coding and thematic extraction using both deductive and inductive methods [30]. Initial coding framework was developed based on a priori themes derived from the interview guide and preliminary themes emerging from the initial interviews. The data were then coded by two members of the research team. During data collection and coding, the research team met regularly to discuss new themes that emerged and refined the coding framework to fully account for participant experiences. Themes were interpreted through a risk environment framework to emphasize within our findings how contextual factors shaped access to SSPs [31, 32]. Of particular interest were how features of the risk environment influenced engagement with housing-based SSPs. While data collection had been suspended due to the pandemic, we determined that we had reached thematic saturation with 26 interviews, as is consistent with norms in qualitative research [33].

## Results

Among a total of 26 participants (see Table 2 for sample characteristics), 12 participants were living in housing facilities that operated an SSP (see Table 3) and one participant had previously lived in such housing. Among the 14 participants who were not living in housing facilities with an SSP, six participants reported procuring injecting equipment through housing-based SSPs. Participant accounts demonstrated how housing-based SSPs improved coverage of injecting equipment by being responsive to PWID’s everyday geographies. If participants had access to sharps disposal containers in their housing, they disposed of their injecting equipment there. Otherwise, they were able to safely dispose of their injecting equipment elsewhere given the wide distribution of harm reduction programming in our setting, with significant coverage of sharps disposal containers.

**Table 2:** Background characteristics of sample (*n* = 26)

	Total	(%)
*Age*		
30–39	4	15
40–49	8	31
50–59	12	46
60–69	2	8
*Gender*		
Man	13	50
Woman	11	42
Two-spirit	2	8
*Race/ethnicity*^a^		
Indigenous	12	46
White	14	54
*HIV status*		
Positive	12	46
Negative	14	54
*Housing status*		
Apartment	5	19
House	1	4
SRO hotel (privately owned)	2	8
SRO hotel (publicly owned)	7	27
Supportive housing	8	31
Shelter	2	8
Friend’s place	1	4
*Drug use in past 30 days*^*a*^		
Cannabis	13	50
Cocaine	11	42
Crack cocaine	13	50
Crystal methamphetamine	11	42
Heroin	23	88
Fentanyl^b^	14	54
Opioids (extra medical)	9	35
Other	4	2

^a^Participants could report multiple categories

^b^Most street-based drugs sold as heroin in Vancouver contain fentanyl

**Table 3:** Housing-based SSPs (*n* = 12)

Housing status		
SRO hotel (publicly owned)	4	33
Supportive housing	7	58
Shelter	1	8

### Distance to programs and operating hours

Most participants injected where they lived, or where their partners and friends lived, as they felt that it was safer and preferred to use drugs in these environments than in public or semi-public spaces, and housing-based SSPs ensured injecting equipment was available when and where most needed. Injecting equipment was usually offered in the lobby, often at the front desk, and, in some cases, on every floor of the buildings. While one participant expressed a desire for injecting equipment to be made available on every floor, participants generally had no trouble in accessing them in the lobby. Many participants highlighted the convenience of living in buildings with an SSP. For example, when asked where she usually gets injecting equipment, one participant responded:

Mostly in my building because it’s right there. Why would I go anywhere else? Yeah. I’ve got everything I need there. […] You’ve got to get them from A to B. So I think having access in buildings where people live would be a lot easier. (58-year-old white woman)

One participant experiencing homelessness also described the ease of acquiring injecting equipment through housing-based SSPs because, even if he was not living in housing facilities with an SSP, these buildings were situated within his everyday spatial environment. He described injecting inside housing facilities that offered him temporary shelter (e.g., friends’ residences, “drug houses” or places where people access and use drugs), and injecting equipment were readily available through SSPs in these settings:

I have clean needles all the time. […] Because all these downtown places, they all have doormen [front desk staff] or whatever, and they all have needles. […] We don’t have to go running around looking for a needle. That’s just downstairs at the front door. (56-year-old Indigenous man)

**Fig. 1:**
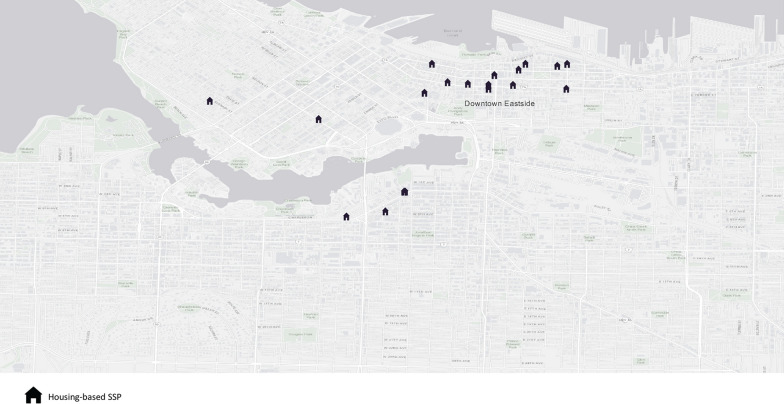
Distribution of housing-based SSPs in Vancouver

In accordance with the distribution of non-profit operated supportive housing and SROs, mapping data showed that housing-based SSPs were concentrated in the DTES (Fig. 1). Several participants whose housing providers did not offer SSPs described traveling to other housing facilities to access these programs. They often preferred to access SSPs in nearby housing facilities rather than traveling a further distance to other settings. One participant described how they and their partner accessed injecting equipment from supportive housing in the DTES while staying at a shelter nearby:

Needles, cookers, yeah. Just wherever we’re close to sometimes, that’s where we go to, and the [supportive housing] is, well it’s because we used to live at [shelter] so that was closest. (44-year-old Indigenous Two-Spirit person)

In addition to sheer distance, participants accessed housing-based SSPs because of their extended hours of operation—typically 24 h a day and 7 days a week. Participants described how housing-based SSPs were often the only reliable SSPs that remained open late into the night. For example, one participant who was not living in a housing facility with an SSP described traveling to a housing-based SSP in the middle of the night to access injecting equipment:

It’s kind of like the last place going this way that I know of is the [supportive housing], which is right here so I mean it’s a 20-minute walk in the middle of the night or 10-minute bus ride. They’re open 24 hours a day. (40-year-old white woman)

### Fear of police

**Fig. 2:**
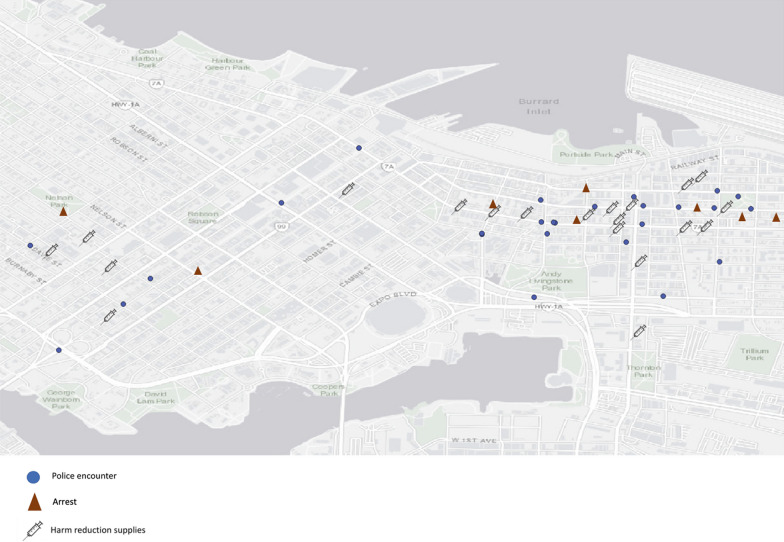
Distribution of police encounters, arrests, and harm reduction services in Vancouver

Many participants preferred to avoid carrying syringes on the street, despite awareness of their legal right to carry syringes. When carrying syringes outside, these participants described doing so discreetly and in limited quantities. As one participant (38-year-old white man) described: *I’m only carrying around a small amount that I need and they fit in a little carrying case or whatever in my backpack.* Concerns over syringe possession were often shaped by previous experiences of being stopped, searched, or arrested by the police—a common practice by local police that disproportionately targets Indigenous people [34]. As part of the mapping exercise, participants were asked to identify locations of police encounters and arrests experienced in their lifetime, if any. Some participants identified multiple police encounters. Mapping data (Fig. 2) revealed that while police encounters and arrests occurred throughout the city, they were concentrated in the DTES and, in particular, close to harm reduction services. One participant who was previously arrested for drug-related charges following an overdose described concerns over accessing injecting equipment at a supervised consumption site (Insite) in the DTES due to the heavy police presence in the surrounding area:

It’s hard because sometimes what if they [police] see me go in, just anybody, and like because they, I’m sure they keep an eye on Insite really. (42-year-old white woman)

Participant narratives revealed how PWID feared that syringe possession could not only be a pretext for criminal charges, but also lead to the confiscation of syringes and drugs by police—a common experience in this setting. For example:

The cops will take them and break them on you. Like they break your pipes and take your rigs [syringes]. […] And not just if they’re used. If they’re brand new they take them. Sometimes they can even charge you with the residue in your rig. (51-year-old white woman)

Participants described how housing-based SSPs addressed these fears by allowing access to injecting equipment while minimizing possible encounters with police. One formerly incarcerated participant recounted experiences of being stopped and searched, describing how moving into a housing facility with an SSP afforded him access that is private and away from the eyes of the police:

Like traveling with needles on you, and if you get stopped by police, they look and they pull it all out, and put it on their trunk […] It’s icky, and I don’t like that part. […] It kind of makes me feel like, I wish there were a better way. And now there is, so. Yeah, it’s really good. Privacy is the major one. (38-year-old Indigenous man)

One participant who was unaware of his right to carry syringes expressed that, because he was concerned about getting arrested due to syringe possession, he was reluctant to carry syringes outside and therefore accessed an SSP within his girlfriend’s housing facility and injected drugs in her unit:

Paraphernalia you can get arrested for, so, I don’t usually carry them on me. […] I don’t want to get arrested. […] I just go to my girlfriend’s building, that way I’m not carrying on me. (40-year-old white man)

### Anti-drug user stigma

For some participants, concerns over unwillingly and unintentionally disclosing their drug use to others hindered access to SSPs outside of housing environments as they would be accessing these services through public spaces. These concerns were rooted in anti-drug user stigma and deterred access to harm reduction services such as supervised consumption sites, as one participant described:

Most people do care. What if these people know I’m using? That’s the only problem I think people have going in there [harm reduction services]. They don’t want other people to see them going in there. Insite and stuff like that. I was like that for years. I didn’t want people to know I was using. (62-year-old white man)

For one participant, the desire to keep his drug use confidential influenced his willingness to access injecting equipment not only from harm reduction services, but also from the HIV service organization he visits regularly:

I don’t know how the people are at [HIV care facility], but I just wouldn’t go in there, because I wouldn’t want them to know that I’m injecting drugs, and that’s all. Yeah, I don’t think it’s really any of their business whether I’m shooting drugs, but that’s how I feel. That’s why I don’t access any harm reduction places, because I just don’t want the public to know what I’m doing. There’s a lot of eyes out there. I just prefer not to let everyone know what I’m doing. […] So just having it in the building is most convenient. (56-year-old Indigenous man)

Housing-based SSPs often offered more private access to injecting equipment, thereby mitigating some risks perceived to be associated with stigmatization. While injecting equipment were often distributed by staff (e.g., receptionist) in housing-based SSPs, participants did not describe concerns about disclosure of drug use in these settings. In line with harm reduction principles, SSPs in non-profit operated supportive housing were often run by staff with lived experience of drug use as well as other intersecting experiences. Participants emphasized how this approach provided more supportive and less stigmatizing environments than settings that were not attended by peer staff. For example, one participant living in women-only supportive housing described how the presence of women staff with lived experience of drug use allowed her to access injecting equipment in a way that is comfortable and not stigmatizing:

It’s safe housing, and they have products there to use, safe injection, if need be. And the women that work there – it’s all women that work there, too – they are very aware of what goes on, and on the streets there. They have experience themselves, so everybody feels comfortable. […] I heard that to get a job there, you have to have had some experience with addictions yourself. […] You don’t have to hide if you’ve got an addiction. You don’t have to hide and have the index finger waved at you. (58-year-old white woman)

## Discussion

This study explored the role of housing-based SSPs in distributing injecting equipment to PWID in Vancouver. PWID preferred to access housing-based SSPs over programs offered at other locations and through other health and social services as they addressed a range of contextual factors: (1) distance to programs and operating hours, (2) fear of police, and (3) anti-drug stigma. Even in a setting with policy commitments to harm reduction, including city-wide syringe distribution programs, we found that access to SSPs remained constrained outside of housing settings for some PWID, and housing-based SSPs served as a critical site within their everyday geographies. Our findings suggest housing-based SSPs as an important approach to improving access to injecting equipment and reducing drug-related harms among PWID.

Distance to SSPs and restrictive operating hours have been widely reported as barriers to access by PWID in Vancouver and other jurisdictions [35]. In many settings, there are few if any services providing access to injecting equipment during non-business hours [11]. While mobile van and mail-based distributions have been established in an effort to address these barriers, both remain limited and are not responsive to immediate needs [18, 36]. Our findings suggest that distance to programs and hours of operation continue to shape access to injecting equipment even in a setting like Vancouver with robust SSPs. We extend existing literature by identifying that housing-based SSPs are responsive to these barriers by cutting travel distance to a minimum and providing extensive hours of operation. Given that many PWID live and/or use drugs in non-profit operated supportive housing and SROs, and many of these housing facilities are staffed 24/7, these settings offer a critical and effective way to enhance SSP coverage.

Fear of police has repeatedly been reported to restrict access to SSPs and other harm reduction programs, even in settings such as ours where syringe possession is legal [37–41]. Our findings suggest that fear of police continues to impede access to SSPs, particularly in heavily policed areas such as the DTES, and sheds light on how PWID utilize housing-based SSPs as a strategy to navigate police presence and practices in their everyday lives. Changes at the legislative level, such as legalization of syringe possession and decriminalization of personal drug possession, are important and necessary steps in transforming the risk environment of PWID. However, these changes alone might not translate into improvements in the lives of PWID subjected to routine police harassment and multiple forms of criminalization, especially those who are racialized. Until meaningful changes to street-level police practices (e.g., harassment, unlawful and racist stop and frisk practices) are made, future SSP programming should consider implementation and scale up of housing-based SSPs to reduce harms among PWID, particularly for those who are disproportionately targeted by police, including Black, Indigenous, and people of color, transgender and gender diverse people, and people living in poverty.

Consistent with existing literature [42, 43], we found that anti-drug stigma and related fear of disclosing drug use hindered access to SSPs in public and semi-public spaces, including in harm reduction and HIV service organizations. Importantly, we also found that integrating SSPs in housing settings minimized drug-related stigma by providing PWID with more discreet access to injecting equipment. Previous research linking stigma and access to harm reduction services have called for efforts to address anti-drug stigma at the individual and structural levels [42]. Despite this, our findings suggest that stigma associated with drug use remains a concern for PWID and continues to restrict access to harm reduction services. Certain sub-populations of PWID, such as women who inject drugs [44], are particularly affected by anti-drug stigma, and its impact on access to SSPs warrants special attention. Until anti-drug stigma is eliminated, housing-based SSPs, particularly those attended by peer staff, should be offered to promote SSP access for PWID.

This study has limitations. First, while we aimed to recruit a diverse sample of participants, their experiences may not be reflective of all PWID. Second, while Indigenous people are disproportionately targeted by police in our setting, our sample size was not sufficiently large to fully characterize these dynamics. Further work is needed to examine differential experiences of everyday lives and geographies within a population of PWID. Finally, data for this study were derived from a parent study examining access to harm reduction supplies and services, inclusive of but not specific to housing-based SSPs. Thus, we did not gather detailed information on the specifics of the various designs of housing-based SSPs and instead focused more broadly on the role of these programs in the everyday lives of PWID. Future research should examine how housing-based SSPs are funded and implemented, and the barriers and facilitators to program uptake and utilization. Despite limitations, our findings draw attention to a previously understudied topic within the harm reduction literature and highlight areas for future research to inform SSP policy and programming decisions.

## Conclusions

The current study advances understanding of access to injecting equipment in a setting with city-wide syringe distribution programs. Our findings underscore the benefits of housing-based SSPs and encourage the expansion of such services to maximize access to harm reduction supports for PWID.

## Data Availability

Ethical restrictions prohibit the authors from making the raw data (full interview transcripts) publicly available as they contain potentially identifying or sensitive participant information. However, all relevant data have been presented within the paper and are fully sufficient to replicate the study findings.

## Abbreviations

PWID: People who inject drugs
SSPs: Syringe services programs
SROs: Single-room occupancy hotels
VANDU: Vancouver Area Network of Drug Users
HCV: Hepatitis C
ACCESS: AIDS Care Cohort to evaluate Exposure to Survival Services
V-DUS: Vancouver Drug Users Study
DTES: Downtown Eastside
